# Predictors of Mid-Term AVNeo Insufficiency

**DOI:** 10.21470/1678-9741-2022-0370

**Published:** 2023-08-07

**Authors:** Vladlen Bazylev, Dmitry Tungusov, Artur Mikulyak

**Affiliations:** 1 Federal Center for Cardiovascular Surgery (Penza), Ministry of Health of the Russian Federation, Penza, Russian Federation

**Keywords:** Aortic Valve Diseases, Aortic Valve Insufficiency, Pericardium, Reoperation, Cardiac Surgical Procedures

## Abstract

**Introduction:**

Aortic stenosis (AS) is the most common valvular heart disease and the most common indication for aortic valve replacement in adults. Aortic valve neocuspidization (AVNeo) with fixed autologous pericardium, also known as the Ozaki procedure, is a possible alternative treatment of AS. Autopericardial valves save the dynamics and physiological anatomy of the aortic root, however, the service life of autopericardial leaflets is limited. There is no data about factors that may influence the development of AVNeo insufficiency. Here, we assessed the effect of autopericardial leaflet symmetry on the development of aortic insufficiency after Ozaki procedure.

**Methods:**

This study included 381 patients with AS who underwent Ozaki procedure. Patients were divided into group 1 (171 patients with symmetric aortic root) and group 2 (210 patients with asymmetric aortic root).

**Results:**

The maximum observation period was up to 65 months. Sixteen cases of aortic insufficiency were detected in group 1, and 33 cases were detected in group 2. Based on the results of Cox regression, the predictors of aortic insufficiency in the late postoperative period are age and asymmetry of neocusps. According to results of Kaplan–Meier analysis, insufficiency of AVNeo in the maximum follow-up period after surgical correction of AS for group 1 patients was significantly lower than for group 2 patients (*P*=0.006).

**Conclusion:**

Asymmetric neocusps increase the risk of aortic insufficiency in the mid-term period after Ozaki procedure. And the older the patients at the time of surgery, the less likely they develop AVNeo insufficiency.

## INTRODUCTION

### Predictors of Mid-Term Aortic Valve Neocuspidization Insufficiency

Aortic stenosis (AS) is the most common valvular heart disease and the most common indication for aortic valve replacement in adults. Aortic valve neocuspidization (AVNeo) with fixed autologous pericardium, also known as the Ozaki procedure, is a possible alternative treatment of AS. The procedure, described by Shigeyuki Ozaki in 2011, entails removing the diseased cusps of the native aortic valve, measuring the intercommissural distance, and shaping new aortic valve cusps from the patient’s autologous pericardium that has first been fixed with glutaraldehyde^[1]^. The AVNeo has better haemodynamic properties and protects the patient from complications associated with patient-prosthetic mismatch, bleeding, and stroke^[2]^. Autologous pericardium elicits lower inflammation and immune response than heterologous pericardium, and it is considered a better material for patches and valve substitutes in cardiovascular surgery^[3]^. In contrast with mechanical and biological valves, autopericardial valves save the dynamics and physiological anatomy of the aortic root. However, the service life of autopericardial leaflets is limited. The author himself, Professor S. Ozaki, claims the development of aortic insufficiency in 7.3% of patients over a follow-up period close to 10 years^[4]^. There is no data about factors that may influence the development of AVNeo insufficiency. In our study, we assessed the effect of autopericardial leaflets symmetry on the development of aortic insufficiency after Ozaki procedure. The objective of this study is to identify predictors of AVNeo regurgitation.

## METHODS

**Fig. 1:**
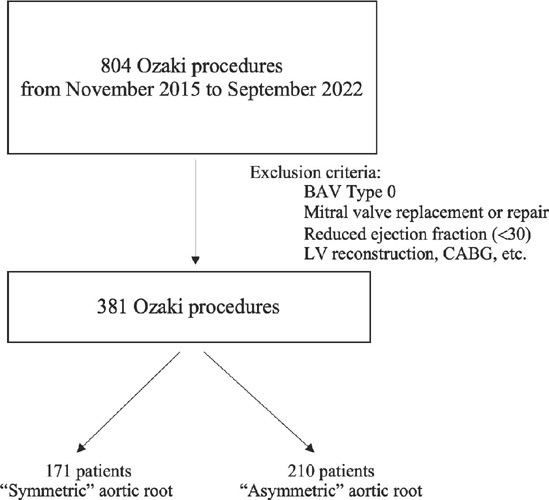
Schematic presentation of research design. BAV=bicuspid aortic valve; CABG=coronary artery bypass grafting; LV=left ventricular.

During the period from November 2015 to September 2022, 804 Ozaki procedures were performed in our medical institution. Baseline characteristics, procedural data, and results were collected prospectively and stored in an electronic database as part of a long-term study of the Ozaki procedure results. The study was approved by the local ethics committee (FCCVS LEC n. 32). Written informed consent was obtained from all patients. Patients with concomitant surgical procedures (mitral valve replacement, etc.) were excluded from the study. Patients who were operated on for moderate or severe aortic valve insufficiency were also excluded. Schematic presentation of research design is shown on the Figure 1. Data collection was carried out at the hospital stage, in the period of 6 to 12 months after surgery, followed by an annual examination and registration of data for up to 65 months (24 [19;35]). This study included 381 patients with AS (aortic valve area < 1 cm², medium transaortic gradient > 40). All patients underwent Ozaki procedure and were divided into two groups:

- Group 1: 171 patients with a symmetric aortic root (all neocusps of the same size).
- Group 2: 210 patients with asymmetric aortic root (at least one of the neocusps differed by at least one size).

The “symmetry” of the aortic root is based only on the size of the aortic valve leaflets. We use the original Ozaki template ranging from 13 to 35. The most frequent templates for our population are 27 to 33. Thus, if the size of all leaflets after measurement is the same (*e.g.*, left coronary cusp, 27; right coronary cusp, 27; non-coronary cusp, 27), we considered the aortic root to be symmetrical. If at least one of them was larger or smaller, we considered the aortic root to be nonsymmetric.

The patients’ clinical and demographic characteristics are presented in Table 1. Both groups are comparable with respect to baseline characteristics.

**Table 1:** Patients’ clinic, demographic, and echocardiographic characteristics.

Parameters	n = 171 (m±SD)	n = 210 (m±SD)	*ʃ*-value
Age, years	59±12	58±12	0.1
Male, n	88 (51%)	113 (53.8%)	0.12
Obesity, n	13 (7.6%)	10 (4.7%)	0.2
BSA, m^2^	1.98±0.28	1.92±0.33	0.05
BMI, kg/m^2^	29.8±4.4	28.9±4.3	0.04
Arterial hypertension	45 (26%)	36 (17%)	0.02
Diabetes, n	24 (14%)	44 (21%)	0.07
Renal failure, n	4 (2.3%)	3 (1.4%)	0.5
6MWT	311.78±54.6	316.66±56.6	0.3
Endocarditis	5 (2.9%)	7 (3.3%)	0.8
NYHA III/IV	29 (17%)	23 (11%)	0.08
EuroSCORE I	5.2±0.9	5.5±1.1	0.2
EuroSCORE II	3.1±0.5	3.2±0.8	0.15
**Echocardiographic parameters**			
EDVs, ml	148±32	145±30	0.07
ESVs, ml	81±15	79±12	0.1
SVs, ml	64±14	65±11	0.06
EFs, %	43±9	44±9	0.08
LV mass, g	332±111	329±106	0.78
Gmax, mmHg	96±21	94±15	0.08
Gmean, mmHg	64±17	62±15	0.09
AVA, sm^2^	0.9±0.2	0.87±0.1	0.06
AVAi, sm^2^	0,45±0,1	0,45±0,1	0.99
EDVIs, ml/m^2^	77±17	78±16	0.05
ESVIs, ml/m^2^	41±8	41±6	0.1
SVIs, ml/m^2^	32.3±6	33±4	0.1
Aortic annulus, mm	22.3±2.6	22.7±3.1	0.1
Aortic annulus £ 21 mm	58 (34%)	65 (31%)	0.63
Sinus of Valsalva, mm	35.8±7.4	34.9±6	0.3
STJ, mm	31.7±7	30.9±5.5	0.2
Ascending aorta, mm	38.8±7.6	38.3±7.8	0.9
Ascending aorta ≥ 40 mm	54 (31%)	65 (30%)	0.68

AVA=aortic valve area; AVAi=indexed aortic valve area; BMI=body mass index; BSA=body surface area; EDV=end-diastolic volume; EDVI=end-diastolic volume index; EF=ejection fraction; ESV=end-systolic volume; ESVI=end-systolic volume index; EuroSCORE=Eu-ropean System for Cardiac Operative Risk Evaluation; Gmax=maximum transaortic gradient; Gmean=medium transaortic gradient; LV=left ventricular; m=mean; 6MWT=6-minute walk test; NYHA=New York Heart Association; SD=standard deviation; STJ=sinotubular junction; SVI=stroke volume index; SV=stroke volume

### Echocardiography

All patients underwent complex echocardiography using ultrasound devices (GE VIVID 7 PRO, VIVID 9, Norway). Transthoracic echocardiographic assessment of the studied parameters in patients was carried out in the heart rate range of 60–80/min and after normalisation of blood pressure (< 150 and 90 mmHg). Echocardiographic measurements were performed in accordance with the recommendations of the American and European Echocardiographic Societies^[5]^.

Transthoracic ultrasound examinations were carried out before surgery. We also performed transesophageal echocardiography during surgery for assessment of neocuspidization quality. Repeat examinations were conducted at the time of hospital discharge, in the period of 6 to 12 months after surgery, followed by an annual assessment and registration of data.

### Surgical Methods

One surgeon performed most of the operations. Surgery was performed through median sternotomy (or upper “J” sternotomy in several cases), with aortic and bicaval cannulation, normothermic perfusion, and antegrade cardioplegia with the use of Custodiol® cardioplegic solution. Only autologous pericardium was used. After median sternotomy, the pericardium was harvested and placed on a metal plate, then it was exposed in 0.6% glutaraldehyde solution for 10 minutes and rinsed three times using physiologic saline solution for six minutes. After aortic cross-clamping and antegrade insertion of the cardioplegic solution, AV was visualised through the transverse aortic incision. The Ozaki procedure was performed after complete aortic root decalcification and measurement of the sinus of Valsalva and aortic annulus with standard templates. The new leaflet was cut out from autologous pericardium and then sutured with running 4-0 monofilament stitches to the native annulus with the smoother (inner) surface of pericardium facing the ventricular side. The commissural coaptation was secured with additional 4-0 monofilament sutures together with a felt pledget at the commissure site outside the aorta. We performed ascending aorta prosthesis in cases of aortic extension (> 45 mm) with Dacron® vascular prosthesis (graft size 26-32 mm).

### Statistical Analysis

The continuous and categorical variables were expressed as mean and standard deviation and counts (percentages), respectively. Period of observation was exposed as median and 1^st^ and 3^rd^ quartiles. Continuous data were analysed using the *t*-test for paired data. The categorical variables were compared using chi-squared test, as appropriate. The cumulative probability of AVNeo insufficiency was estimated by the Kaplan–Meier method. Logrank tests were used to compare both groups. Cox regression was used to investigate the effect of clinical variables upon the time on AVNeo insufficiency. Results were reported as hazard ratios (HRs) with the 95% confidence interval of probability values. For all statistical tests, a *P*≤0.05 was considered significant. All statistical analyses were performed with IBM Corp. Released 2017, IBM SPSS Statistics for Windows, version 25, Armonk, NY: IBM Corp.

## RESULTS

Intraoperative and early echocardiographic characteristics of both groups of patients are presented in Table 2. None of the patients had aortic valve insufficiency in the early postoperative period. The main intraoperative and echocardiographic characteristics were also comparable.

**Table 2:** Patients’ intraoperative and early postoperative characteristics.

Parameters	n = 171 (m±SD)	n = 210 (m±SD)	*ʃ*-value
CPB time, min	126±25	131±27	0.06
Aortic cross-clamping time, min	99±16	102±21	0.12
Ascending aorta replacement	37 (21.6%)	54 (25.7%)	0.42
Days in ICU	1.1±0.8	1.1±0.8	0.99
**Echocardiographic parameters**			
EDVs, ml	134±27	132±28	0.48
ESVs, ml	61.9±13	64.1±14	0.15
SVs, ml	72.2±21	68.2±19	0.06
EFs, %	53±12	51±11	0.09
Gmax, mmHg	11±4.4	10.4±4.5	0.19
Gmean, mmHg	5±3.5	4.2±4.3	0.06
AVA, sm^2^	3.2±1.1	3.3±1	0.35
EDVIs, ml/m^2^	67.7±18	68.7±18	0.59
ESVIs, ml/m^2^	31.3±8	32.8±7.6	0.6
SVIs, ml/m^2^	36.2±6	35.4±5	0.16

AVA=aortic valve area; CPB=cardiopulmonary bypass; EDV=end-diastolic volume; EDVI=end-diastolic volume index; EF=ejection fraction; ESV=end-systolic volume; ESVI=end-systolic volume index; Gmax=maximum transaortic gradient; Gmean=medium transaortic gradient; ICU=intensive care unit; M=mean; SD=standard deviation; SV=stroke volume; SVI=stroke volume index

The maximum observation period was up to 65 months (24 [19;35]).

Echocardiography data in the mid-term period are shown in Table 3. All patients showed an increase in cardiac contractility and decrease in left ventricular volume and myocardial mass. However, the sizes of the left ventricle were larger in the second group. During the indicated observation period, 16 cases of aortic insufficiency were detected in group 1, and 33 cases of aortic insufficiency were detected in group 2. Cox regression was performed to identify predictors of aortic insufficiency recurrence. The results are presented in Table 4.

**Table 3:** Patients’ echocardiographic characteristics (mid-term follow-up).

Parameters	n = 171 (m±SD)	n = 210 (m±SD)	*P*-value
EDV, ml	117±25	124±29	0.01
ESV, ml	47.6±16	53.1±18	0.01
SV, ml	69.7±13.5	71±14	0.36
EF, %	60.8±12.5	58±11.5	0.02
LV mass, g	263±71	272±84	0.26
Gmax, mmHg st.	13±5	12.5±5	0.33
Gmean, mmHg st	6.2±3.2	6.0±2.6	0.5
AVA, sm²	2.9±0.9	3.1±1.1	0.06
AVAi, sm²/m²	1.5±0.5	1.6±0.5	0.06
EDVIs, ml/m^2^	60.2±12	64±14	0.01
ESVIs, ml/m^2^	24±8	27±9	0.01
SVIs, ml/m^2^	35±9	37±12	0.07
AV insufficiency (moderate and severe)	16 (9%)	33 (15.7%)	0.04

AV=aortic valve; AVA=aortic valve area; AVAi=indexed aortic valve area; EDV=end-diastolic volume; EDVI=end-diastolic volume index; EF=ejection fraction; ESV=end-systolic volume; ESVI=end-systolic volume index; Gmax=maximum transaortic gradient; Gmean=medi-um transaortic gradient; LV=left ventricular; M=mean; SD=standard deviation; SV=stroke volume; SVI=stroke volume index

**Table 4:** Results of Cox regression.

Predictors	Multiple Cox regression			Univariate Cox regression		
	HR	95% CI	*P*-value	HR	95% CI	*P*-value
Age	0.9	0.9-1	0.1	0.9	**0.9-1.1**	**0.02**
Male	0.5	0.2-1.1	0.1	0.5	0.3-1	0.06
Diabetes	0.3	0.1-0.3	0.1	2.3	0.8-6.5	0.1
Aortic annulus, mm	1.01	0.9-1.1	0.8	1.07	0.9-1.1	0.07
Endocarditis	5.2	jun.-41	0.1	2.2	0.3-16	0.4
**Asymmetry**	**2.6**	**1.3-5**	**0.003**	**2.2**	**1.2-4.1**	**0.007**

CI=confidence interval; HR=hazard ratio

Based on the results of Cox regression, the predictors of aortic insufficiency in the late postoperative period are age (univariant Cox regression, HR 0.9; *P*=0.02) and asymmetry of neocusps (multiple Cox regression, HR 2.6; *P*=0.03).

**Fig. 2:**
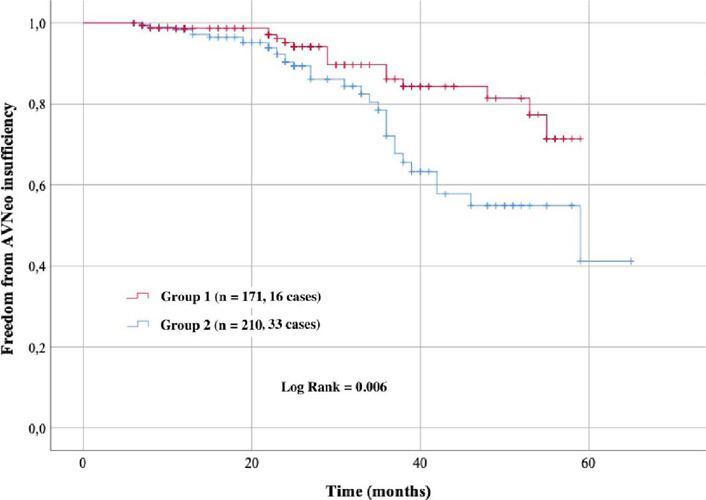
Cumulative probability of aortic valve neocuspidization (AVNeo) insufficiency (Kaplan–Meier method).

Results of Kaplan-Meier analysis are shown in Figure 2.

Insufficiency of AVNeo in the maximum follow-up period after surgical correction of AS for patients with symmetric neocusps was significantly lower than in the group of patients with asymmetric neocusps (*P*=0.006).

**Fig. 3:**
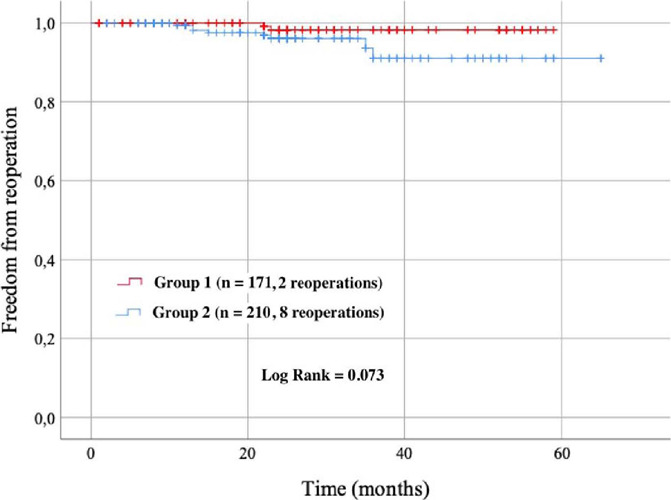
Cumulative probability of aortic valve neocuspidization (AVNeo) reoperation (Kaplan–Meier method).

During the observation period, two patients have been reoperated on in the 1st group, and eight in the 2nd group. Results of KaplanMeier analysis are shown in Figure 3.

## DISCUSSION

Aortic valve replacement with a mechanical or biological prosthesis is the gold standard for treatment of aortic valve pathology^[5,6,7]^. The Ozaki procedure aims to reconstruct the aortic valve in patients with AS, aortic regurgitation, and infective endocarditis. This procedure has a number of advantages, such as satisfactory hemodynamic parameters, as well as the absence of the need for anticoagulant therapy in the postoperative period^[4,6,8,9]^. Also, AVNeo reduces the risk of complications and patient-prosthetic mismatch^[6,10,11]^. In the current literature, the number of studies on the symmetry of the aortic valve leaflets is limited. Symmetry or proportionality of the autopericardial valves implies the equal intercommissural distance and height of the leaflets. According to a study by Becker and Vollebergh, the difference in leaflet sizes is the cause of uneven mechanical loading, leading to valve degradation^[11]^. Symmetry is an important factor in preventing the development of aortic regurgitation. We hypothesize that the advantage of symmetrical aortic valve leaflets in the Ozaki procedure is that they are more resistant to fluctuations in hydrodynamic pressure. According to the study by S. Ozaki, the symmetric tricuspid aortic valve has the best hemodynamic characteristics and the most correct anatomical and physiological configuration^[12,13,14]^.

The second predictor of AVNeo failure was the age of the patient. S. Ozaki has described the first series of 404 patients in 2014^[14]^. In that study, the patients were older than in our research (69.0±12.9 years *vs.* 59±12 years). The second, subsequent study has included 850 patients with a mean age of 71years. Ozaki described a rate of freedom from reoperation of 96.2% after 53 months in the first series, which is comparable with our results of 97.4% up to 65 months of follow-up. The cumulative incidence for reoperation was 4.2% in 850 patients over a 10-year period, which depicts an exciting option for the treatment of aortic valve pathologies^[15]^. Ozaki procedure is a kind of alternative to biological aortic valve replacement. Bourguignon et al.^[16]^ reported their single-center long-term experience using the Edwards Perimount valve for aortic valve replacement in 2600 patients. The 10-, 15-, and 20-year freedom from reoperation was 93.2±0.8%, 81.5%±1.9%, and 54.3%±4.8%, respectively. Bourguignon et al.^[16]^ have showed a clear association between younger age and shorter durability of biological prostheses. Freedom from reoperation in the age class below 60 years significantly decreases at 15 and 20 years to 70.8±4.1 and 38.1%±5.5%, respectively. It can be assumed that the decreased durability in younger age is also true for the AVneo procedure.

Of course, the Ozaki procedure has advantages and disadvantages. On the one hand, this is a longer surgery and there is a longer aortic cross-clamping time; on the other hand, these are excellent hemodynamic properties comparable to a native aortic valve and no need for anticoagulation therapy. In our study, the incidence of AVNeo regurgitation is quite significant, but freedom from reoperation is only 97.4%. If clear indications for this procedure are formulated, the frequency of AVNeo insufficiency and reoperations can be reduced to a minimum. In our study, we found two predictors — symmetry of aortic root and age of the patient. Thus, the Ozaki procedure can be indicated for older patients. At the same time, freedom from reoperation and the development of aortic insufficiency will be significantly lower in patients with a symmetrical aortic root. Further study of the results of AVNeo will allow us to answer the question more accurately in what cases this surgery will have advantages.

## CONCLUSION

1. Asymmetric neocusps increase the risk of aortic insufficiency in the mid-term period after the Ozaki procedure.
2. The older the patients at the time of surgery, the less likely they develop AVNeo insufficiency.

## Abbreviations, Acronyms & Symbols

AS: Aortic stenosis
AV: Aortic valve
AVA: Aortic valve area
AVAi: Indexed aortic valve area
AVNeo: Aortic valve neocuspidization
BAV: Bicuspid aortic valve
BMI: Body mass index
BSA: Body surface area
CABG: Coronary artery bypass grafting
CI: Confidence interval
CPB: Cardiopulmonary bypass
EDV: End-diastolic volume
EDVI: End-diastolic volume index
EF: Ejection fraction
ESV: End-systolic volume
ESVI: End-systolic volume index
EuroSCORE: European System for Cardiac Operative Risk Evaluation
Gmax: Maximum transaortic gradient
Gmean: Medium transaortic gradient
HR: Hazard ratio
ICU: Intensive care unit
LV: Left ventricular
6MWT: 6-minute walk test
M: Mean
NYHA: New York Heart Association
SD: Standard deviation
STJ: Sinotubular junction
SV: Stroke volume
SVI: Stroke volume index
